# Feasibility, procedural efficiency, and early imaging outcomes of concomitant pulsed field ablation and left atrial appendage closure: a prospective single-centre study

**DOI:** 10.1093/europace/euag017

**Published:** 2026-02-06

**Authors:** Brandon Doty, Mohamed Mraiyan, Ganesh Nair, Momin Khan, Kirollos Gabrah, Devi G Nair

**Affiliations:** Arrhythmia Research Group, Jonesboro, AR, USA; Arrhythmia Research Group, Jonesboro, AR, USA; St. Bernards Medical Center, 201 E Oak Ave, Jonesboro, AR 72401, USA; Arrhythmia Research Group, Jonesboro, AR, USA; Arrhythmia Research Group, Jonesboro, AR, USA; Arrhythmia Research Group, Jonesboro, AR, USA; Arrhythmia Research Group, Jonesboro, AR, USA; St. Bernards Medical Center, 201 E Oak Ave, Jonesboro, AR 72401, USA

**Keywords:** Atrial fibrillation, Pulsed field ablation, Left atrial appendage closure, Concomitant intervention, Imaging outcomes, Real-world cohort

## Abstract

**Aims:**

Concomitant pulsed field ablation (PFA) for atrial fibrillation (AF) with left atrial appendage closure (LAAC) offers a single-procedure approach for arrhythmia control and thromboembolic risk reduction. This study assessed the workflow, safety, and feasibility of combined PFA and LAAC in routine practice.

**Methods and results:**

We prospectively analysed patients undergoing zero-fluoroscopy PFA, with low fluoroscopy for LAAC. Pre-procedural planning used CT imaging and AI-based models for device selection and landing-zone assessment. A single transseptal puncture facilitated intracardiac echocardiography, PFA catheter, and LAAC sheath. A total of 209 patients were included (56% male; mean age 76.5 ± 7.8 years), with 59.3% paroxysmal AF, 40.7% persistent AF, and 50% *de novo* AF. The mean CHA_2_DS_2_-VASc score was 4.5. Mean procedure and left atrial dwell times were 57.3 ± 17 and 45.1 ± 13.6 min, respectively; fluoroscopy averaged 3.4 ± 0.8 min for LAAC. A single LAAC device was used in 94% of cases, achieving adequate seal in all. No pericardial effusion, phrenic nerve injury, kidney, or oesophageal injury occurred; two patients had minor groin bleeding. All were discharged same day on oral anticoagulation for 90 days. Follow-up CT (80%) or TEE (20%) at 111.6 ± 16.5 days showed no leaks >2 mm, a 4.7% small-leak rate, and two device-related thrombi without stroke, managed with extended anticoagulation.

**Conclusion:**

Combined PFA and LAAC is feasible and safe with favourable early outcomes. Multi-centre studies are warranted to confirm findings and standardize this workflow for broader clinical adoption.

## Graphical Abstract

**Graphical Abstract:**
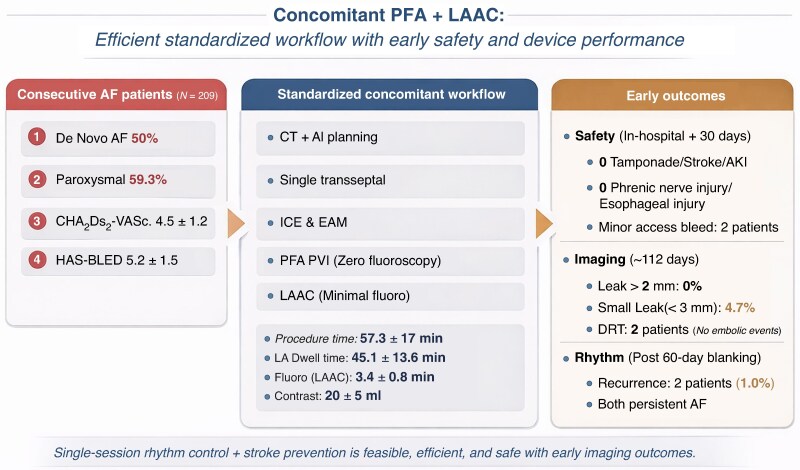

What’s new?

- This is one of the largest prospective real-world cohorts evaluating a standardized concomitant workflow for pulsed PFA and LAAC performed during the same procedure.
- A single transseptal, low fluoroscopy strategy using pre-procedural CT planning enabled highly efficient combined therapy with excellent procedural success and low complication rates.
- Concomitant PFA + LAAC demonstrated high acute appendage seal rates, with low rates of clinically significant peri-device leak and rare device-related thrombus on follow-up imaging.
- Rhythm outcomes following integrated therapy showed favourable early rhythm observations, supporting the feasibility of an integrated ‘one-stop’ AF care model incorporating stroke prevention and rhythm control.

## Introduction

Atrial fibrillation (AF) remains the most common sustained arrhythmia worldwide and is associated with substantial morbidity, including impaired quality of life, heart failure exacerbation, and increased risk of thromboembolic stroke.^1,2^ Catheter ablation is an established rhythm control strategy for symptomatic patients and is increasingly emphasized earlier in the disease course, supported by contemporary guideline frameworks promoting integrated AF management.^1^ However, many patients with AF also have elevated bleeding risk, contraindications to long-term oral anticoagulation (OAC), or experience adverse events related to anticoagulant therapy. In these populations, percutaneous left atrial appendage closure (LAAC) has emerged as an effective alternative strategy for stroke prevention.^3,4^

Importantly, patients referred for AF ablation and LAAC frequently represent an overlapping cohort, creating an opportunity to integrate rhythm control and stroke prevention within a single procedural encounter. Concomitant procedures may offer advantages including avoidance of repeat transseptal access, reduced cumulative anaesthesia exposure, shortened total hospitalization time, and improved patient-centred efficiency. However, combined ablation and LAAC has historically raised concerns regarding procedural complexity, incremental risk of pericardial effusion, thrombus formation, and challenges in optimal device seating or seal in the setting of acute atrial oedema following ablation. Early single-centre experiences suggest feasibility of concomitant pulsed field ablation (PFA) and LAAC, but real-world data on standardized workflows and early imaging outcomes remains limited.^5^

PFA is an emerging nonthermal ablation modality that delivers myocardial-selective electroporation and has demonstrated noninferior efficacy compared with conventional thermal ablation, with a favourable safety profile including reduced collateral injury to surrounding structures.^6–9^ As PFA adoption expands globally, there is increasing interest in leveraging its procedural efficiency and safety to enable integrated structural and electrophysiology workflows, including concomitant LAAC. Yet, data remains limited regarding real-world safety, procedural efficiency, imaging outcomes, and longer-term rhythm outcomes following combined PFA and LAAC, particularly within standardized workflows designed to streamline procedural execution.^5^

Therefore, we evaluated the acute and mid-term safety, procedural characteristics, imaging outcomes, and arrhythmia-related efficacy of a standardized concomitant PFA and LAAC workflow in a large prospective cohort. We sought to assess whether an integrated approach could be delivered safely and efficiently while maintaining high rates of successful appendage closure and durable rhythm control.

## Methods

### Patient population

This was a prospective, non-randomized, single-centre cohort study of consecutive patients with non-valvular AF undergoing concomitant PFA and LAAC at St. Bernards Medical Center between October 2024 and July 2025. All patients were referred for LAAC based on clinical indication (*Table 1*); ablation eligible patients were offered single-session PFA at the time of LAAC as part of a standardized workflow. Baseline demographics, comorbidities, AF classification (paroxysmal vs. persistent), procedural characteristics, and device-related outcomes were collected prospectively. Stroke and bleeding risk scores were calculated using the CHA_2_DS_2_-VASc and HAS-BLED scoring systems. The study was conducted in accordance with the Declaration of Helsinki and was approved by the locally appointed ethics committee (IRB #NCF456D2); informed consent was waived. The data underlying this article will be shared on reasonable request to the corresponding author.

**Table 1:** Baseline characteristics of the study population

Characteristic	Overall (*n* = 209)
Age, years	76.5 ± 7.8
Male sex, *n* (%)	117 (56.0)
De novo AF, *n* (%)	105 (50.0)
AF subtype, *n* (%)	
Paroxysmal AF	124 (59.3)
Persistent AF	85 (40.7)
CHA_2_DS_2_-VASc score	4.5 ± 1.2
HAS-BLED score	5.2 ± 1.5
Comorbidities, *n* (%)	
Hypertension	144 (68.9)
Diabetes mellitus	96 (45.9)
Heart failure	88 (42.1)
CAD/prior MI/PCI/CABG	121 (57.9)
Prior stroke or TIA	92 (44.0)
Chronic kidney disease	65 (31.1)
Vascular disease/peripheral artery disease	46 (22.0)
Bleeding history and anticoagulation profile, *n* (%)	
Prior major bleeding requiring transfusion	86 (41.1)
Prior ICH	29 (13.9)
History of gastrointestinal bleed	75 (35.9)
Clinically relevant non-major bleeding	31 (14.8)
High bleeding risk (includes fall risk, medication interactions)	20 (9.6)
Patient preference	11 (5.3)

Values are presented as mean ± SD or *n* (%). AF, atrial fibrillation; CABG, coronary artery bypass grafting; CRNMB, clinically relevant non-major bleeding; ICH, intracranial haemorrhage; MI, myocardial infarction; PCI, percutaneous coronary intervention; TIA, transient ischaemic attack

### Pre-procedural imaging and planning

**Figure 1:**
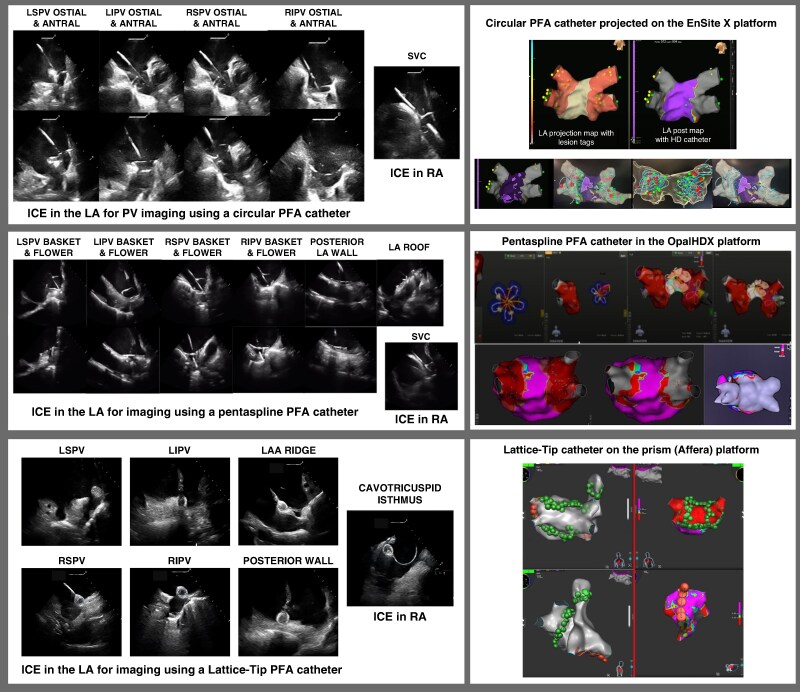
**Pre-procedural imaging and intraprocedural planning workflow for LAAC.** CT and AI-based planning were used when feasible, with intraprocedural TEE/ICE-based sizing in patients with contrast limitations. Panel 1 shows CT 3D reconstruction of the LAA used for anatomical assessment and sizing. Panel 2 demonstrates AI-based modelling to evaluate optimal device size and landing-zone position. Panel 3 shows intraprocedural TEE and ICE imaging used to guide device positioning and confirm seal criteria. Panel 4 shows post-ablation remeasurement of the LAA prior to final device sizing and deployment. This workflow supported standardized sizing and imaging-confirmed device deployment.

Pre-procedural computed tomography (CT) imaging with artificial intelligence (AI)-based planning using FEops HEARTguide was performed in 144 patients (69%) to support LAAC device sizing, landing-zone assessment, and procedural strategy planning; the software provides patient-specific 3D left atrial appendage (LAA) reconstruction with predicted landing-zone diameter/depth and recommended device size/positioning (*Figure 1*). CT was not performed in the remaining patients due to chronic kidney disease and the need to avoid iodinated contrast; in these patients, intraprocedural transoesophageal echocardiography (TEE) was used for appendage assessment and device sizing. A subset of implants (22%) were performed under three-dimensional (3D) intracardiac echocardiography (ICE) guidance, and all patients in this subgroup underwent pre-procedural CT planning. The present analysis was not designed to assess concordance between AI recommendations and operator-selected device size or to evaluate the independent impact of AI planning on procedural or imaging outcomes.

### Periprocedural hydration and volume management

A standardized hydration protocol was used given the advanced age and high-risk profile of the cohort, including patients with chronic kidney disease. Patients received 500 mL of normal saline pre-procedure and 500 mL intra-procedure, with additional volume management guided by intraprocedural haemodynamics. When left atrial pressure exceeded 25 mmHg, a single dose of intravenous diuretic was administered post-procedure at the operator’s discretion.

### Procedure workflow

All procedures were performed using a standardized integrated workflow designed to minimize fluoroscopy exposure and procedural time. ICE and electroanatomical mapping (EAM) were used in all cases, and a single transseptal puncture (TSP) strategy was employed to facilitate ICE imaging, left atrial mapping and ablation, and LAAC device delivery.

### Transseptal access, ICE positioning, and sheath management

A single transseptal puncture (TSP) was used in all cases to facilitate ICE, EAM, PFA, and subsequent LAAC, with no procedures requiring crossover to a second puncture. The TSP site was individualized and intentionally optimized based on AI-based pre-procedural planning and/or intraprocedural imaging to support both ablation and LAAC delivery. Following transseptal access using the system-specific PFA sheath and a pigtail radiofrequency wire, the wire was retained in the left atrium, and the ICE catheter was advanced into the left atrium through the same transseptal site alongside the retained wire prior to ablation. The PFA sheath was subsequently advanced through the same transseptal access for mapping and ablation. After completion of PFA, a single sheath exchange was performed to a 17 Fr steerable LAAC delivery sheath, which facilitated coaxial alignment with the LAA. No additional sheath exchanges, prolonged left atrial access times, or access-related complications attributable to the single-access strategy were observed.

### Anticoagulation strategy during the procedure

Femoral venous access was obtained under ultrasound guidance. Intravenous unfractionated heparin was administered prior to TSP to achieve and maintain a target activated clotting time greater than 350 s throughout the procedure.

### PFA procedure

Under ICE guidance, a single TSP was performed using a transseptal sheath and radiofrequency-enabled pigtail wire. Following transseptal access, the ICE catheter was advanced into the left atrium alongside a steerable sheath through the single transseptal site. Left atrial and pulmonary vein (PV) geometry was acquired using a 3D mapping system with a high-definition mapping catheter.

### PFA systems and mapping platforms

**Figure 2:**
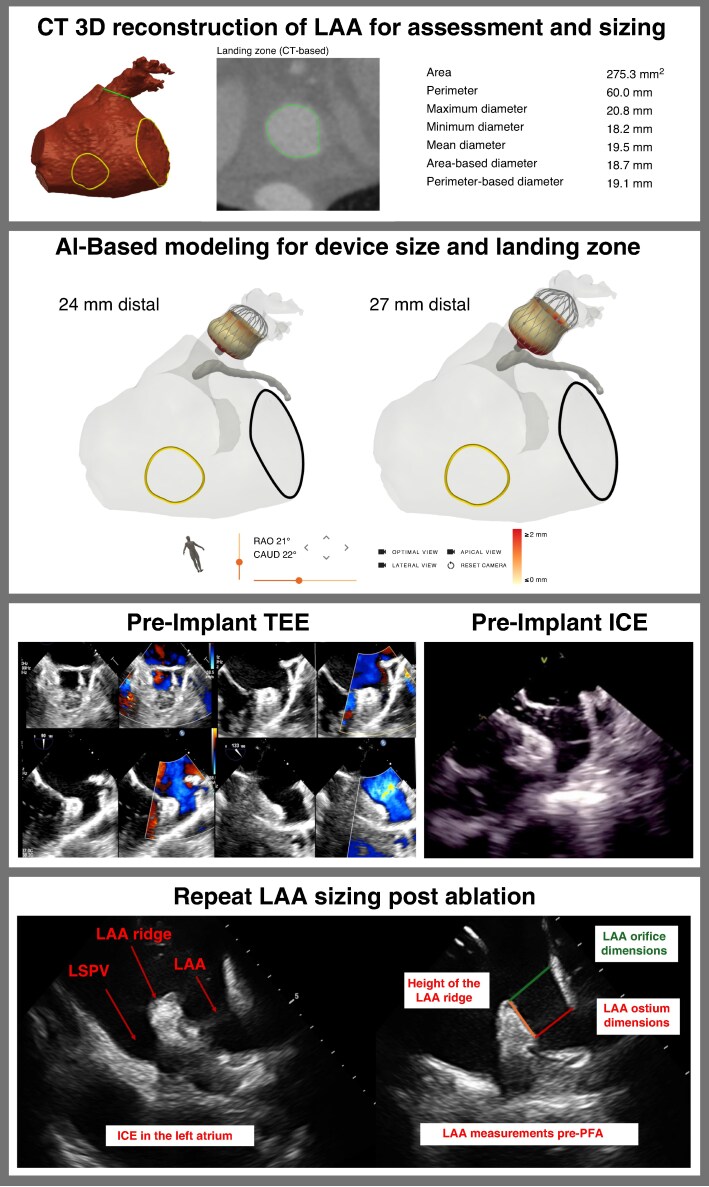
Representative ICE imaging and 3D EAM reconstructions during PFA enabling a zero-fluoroscopy pulmonary vein isolation strategy using different catheter platforms. Left panels show ICE imaging of multiple PFA catheter platforms during LA navigation and ablation, including positioning within the PVs, PW, and SVC. ICE provided real-time confirmation of catheter location and tissue contact, enabling a fluoroscopy-free PFA PVI workflow. Right panels show 3D EAM reconstructions across platforms: a pentaspline PFA catheter on Opal HDx (top), a circular PFA catheter on EnSite X (middle), and a lattice-tip catheter on Prism (Affera) (bottom). Maps depict LA anatomy and PFA lesion delivery for PVI, including PW and adjunctive targets when performed, within a standardized workflow.

Three PFA systems were used in this cohort. The Farapulse (Boston Scientific) catheter with integrated mapping was utilized within the Opal HDx platform in 45% of patients. The PulseSelect (Medtronic) catheter was projected onto the EnSite X platform in 22% of patients. The Affera Sphere-9 (Medtronic) catheter with the Prism mapping platform was used in 33% of patients. Sample maps from each platform are demonstrated in *Figure 2*.

PFA was performed with an ostial-to-antral approach to achieve pulmonary vein isolation (PVI). The ablation catheter was advanced into each PV over a guidewire, and lesion delivery was guided by ICE and 3D mapping to ensure adequate tissue contact and lesion overlap for circumferential coverage (*Figure 2*). Additional antral lesion sets were applied to achieve wide antral isolation. At the completion of ablation, acute PVI was confirmed by entrance and exit block testing. All portions of the ablation procedure were performed without fluoroscopy.

### Adjunctive ablation strategy

Adjunctive ablation beyond PVI was not systematically performed and was undertaken at operator discretion based on clinical indication and intraprocedural findings. Posterior wall (PW) isolation was performed in patients with persistent AF when EAM demonstrated low-voltage substrate involving the PW. Cavotricuspid isthmus (CTI) and mitral isthmus ablation were performed in patients with a clinical history of typical atrial flutter or inducible flutter during the procedure. Superior vena cava (SVC) isolation was performed in all patients except when monopolar PFA was used in the presence of cardiac rhythm management devices, in whom SVC ablation was avoided. Adjunctive lesion sets were predominantly delivered using PFA. CTI ablation was performed using radiofrequency energy when a dual-energy PFA catheter was employed; all other adjunctive ablation applications were performed using PFA only.

### LAAC procedure

Following completion of PFA, the ablation catheter was removed. ICE imaging was used to reassess LAA anatomy, confirm measurements and device planning derived from pre-procedural CT, and evaluate for any acute tissue oedema that could impact device seating. In patients without pre-procedural CT imaging, appendage measurements and sizing were guided by intraprocedural TEE and ICE. Although post-ablation reassessment of the LAA was performed to confirm device seating feasibility, this study was not designed to systematically quantify post-PFA changes in landing-zone dimensions. Device sizing was based on pre-ablation measurements, and devices were not intentionally undersized to account for acute oedema.

**Figure 3:**
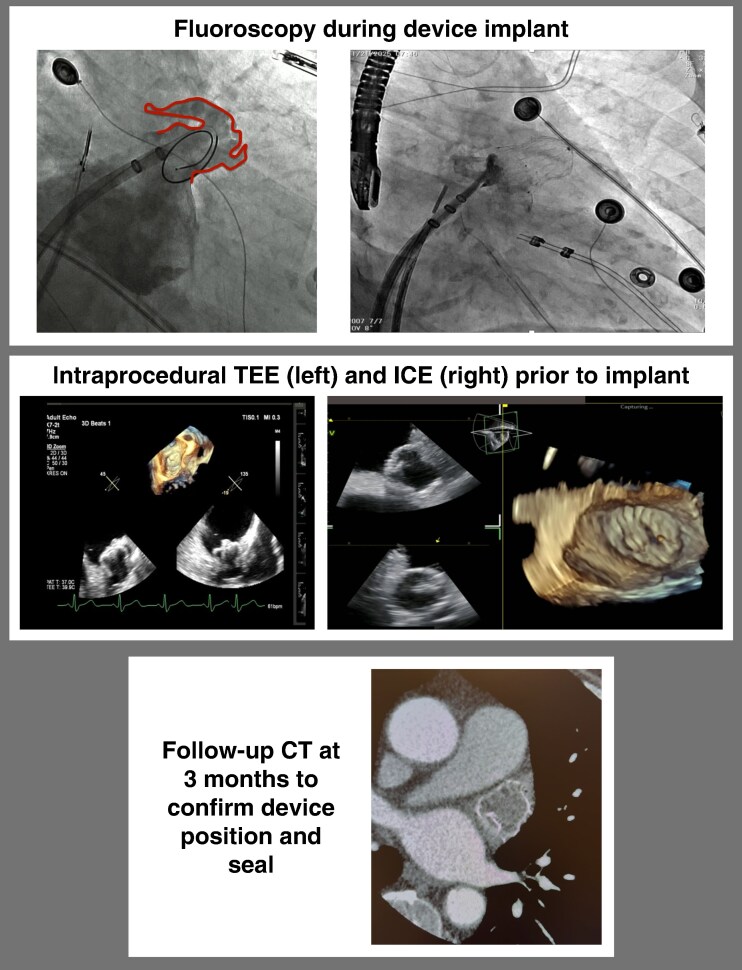
**Intraprocedural and follow-up imaging for LAAC device assessment.** Panel 1 shows fluoroscopic imaging during device implantation. Panel 2 shows intraprocedural TEE and ICE used to confirm device position and assess release criteria prior to implantation. Panel 3 shows follow-up CT imaging at approximately 3 months used to assess device position and peri-device seal; CT was performed in 80% of patients for leak assessment, with TEE used in the remaining 20%.

A pigtail catheter was advanced into the body of the LAA and the LAAC delivery sheath was advanced through the same transseptal access site. The LAAC device was delivered into the predetermined landing zone guided by the pre-procedural planning software and ICE. Minimal fluoroscopy and contrast were used during the LAAC portion of the procedure to confirm angle, depth, and positioning. Device stability was confirmed using a tug test, and peri-device leak was assessed by ICE (including colour Doppler) with contrast angiography as needed (*Figure 3*).

### Post procedural management and follow-up

All patients were discharged the same day on OAC for 90 days following the index procedure. Follow-up imaging with CT or TEE was performed at 90–120 days. Per institutional protocol, CT was the preferred follow-up modality to provide standardized assessment of peri-device leak and device position (*Figure 3*). TEE was used when CT was not feasible or clinically indicated (CT in 80% of patients). Adequate seal was defined as absence of uncovered lobes with peri-device leak <3 mm. OAC was discontinued on the day of follow-up imaging when adequate seal was confirmed, and patients were transitioned to single antiplatelet therapy. If adequate seal was not confirmed, OAC was continued, and repeat imaging and/or subsequent intervention was pursued at the discretion of the treating physician. In the two patients with DRT, OAC was continued and subsequently transitioned to low-dose OAC after repeat imaging demonstrated DRT resolution.

### Study endpoints and definitions

The primary objectives were to evaluate feasibility and safety of the integrated concomitant workflow, including acute procedural success, periprocedural complications, and imaging outcomes at follow-up. Secondary endpoints included procedural efficiency metrics and arrhythmia-related clinical outcomes during follow-up.

### Acute procedural success

Acute procedural success was defined as successful PVI confirmed by entrance and exit block at the end of the ablation procedure, together with successful LAAC device implantation meeting all release criteria (device position, anchoring, compression, and seal). Acute appendage seal was assessed intra-procedurally by ICE with colour Doppler, with angiographic confirmation as needed.

### Periprocedural and early safety outcomes

Periprocedural complications were defined as adverse events occurring during the index hospitalization or within 30 days post-procedure. Safety outcomes included:

- Pericardial effusion/tamponade, defined as pericardial effusion requiring drainage, surgical intervention, haemodynamic support, or resulting in prolonged hospitalization.
- Stroke or transient ischaemic attack (TIA), defined as a new neurologic deficit lasting ≥ 24 h (stroke) or < 24 h (TIA), adjudicated clinically and/or by neuroimaging.
- Major bleeding, defined as bleeding associated with haemodynamic compromise, transfusion, procedural intervention, a decrease in haemoglobin ≥ 2 g/dL, bleeding at a critical site, or resulting in prolonged hospitalization.
- Vascular complications, defined as access-site haematoma requiring transfusion or intervention, pseudoaneurysm, or arteriovenous fistula requiring intervention or any access-related complication resulting in prolonged hospitalization.
- Acute kidney injury (AKI), defined as an increase in serum creatinine ≥ 0.3 mg/dL within 48 h of the procedure or ≥ 50% increase from baseline.
- Phrenic nerve injury, defined as new diaphragm dysfunction detected clinically or by imaging after ablation.
- Oesophageal injury, defined as evidence of oesophageal ulceration, fistula, or clinically suspected oesophageal injury requiring evaluation or intervention.

Minor access-site bleeding events not meeting major bleeding criteria were reported separately.

### Imaging outcomes (device performance)

Follow-up imaging with CT or TEE performed at 90–120 days was used to evaluate device position, peri-device leak, and DRT.

- Peri-device leak was assessed by CT or Doppler imaging and categorized as none (0 mm), small (< 3 mm), moderate (3–5 mm), or large (> 5 mm).
- Adequate seal was defined as absence of uncovered lobes with peri-device leak < 3 mm.
- DRT was defined as a discrete echodensity or filling defect adherent to the LAAC device, distinct from normal post-implant healing tissue, identified on TEE or CT.
- Significant leak was defined as peri-device leak ≥ 3 mm (moderate or large) and prompted continuation of anticoagulation and follow-up management per clinical protocol.

### Arrhythmia-related outcomes

Arrhythmia outcomes were assessed using routine clinical follow-up and available rhythm monitoring modalities (e.g. electrocardiography, ambulatory monitoring, implantable loop recorder when available).

- Arrhythmia recurrence was defined as any documented episode of AF, atrial flutter, or atrial tachycardia lasting more than 30 s occurring after a 60 day blanking period.
- Repeat ablation, cardioversion, or antiarrhythmic drug escalation occurring after the blanking period was recorded as a clinically significant rhythm outcome.

### Procedural efficiency endpoints

Procedural efficiency endpoints included total procedure time (skin-to-skin), left atrial dwell time, fluoroscopy time, and contrast volume during the LAAC portion of the procedure. Same-day discharge was recorded as a procedural care delivery outcome.

### Statistical analysis

Descriptive statistics were used to characterize study outcomes with no performance of hypothesis testing. Continuous variables are reported as mean ± standard deviation, and categorical variables are summarized as counts and percentages.

## Results

### Study population and baseline subject characteristics

A total of 209 consecutive patients with non-valvular AF underwent concomitant PFA and LAAC using a standardized integrated workflow between October 2024 and July 2025. Baseline demographic and clinical characteristics are summarized in *Table 1*. The cohort reflected elevated thromboembolic and bleeding risk, with mean CHA_2_DS_2_-VASc and HAS-BLED scores of 4.5 ± 1.2 and 5.2 ± 1.5, respectively. AF subtype distribution was 59.3% paroxysmal and 40.7% persistent, and 50% of patients had *de novo* AF.

### Procedural characteristics and acute procedural success

Acute procedural success was achieved in 100% of cases, defined as confirmed PVI by entrance and exit block and successful LAAC device implantation meeting all release criteria. Acute appendage seal at the time of implantation was achieved in 100% of cases. Total procedure time was 57.3 ± 17.0 min, and left atrial dwell time was 45.1 ± 13.6 min. Fluoroscopy time during the LAAC portion of the procedure was 3.4 ± 0.8 min, and contrast volume used for LAAC confirmation was 20 ± 5 mL. Same-day discharge was achieved in 100% of patients. Procedural characteristics and acute outcomes are summarized in *Table 2*.

**Table 2:** Procedural characteristics and acute outcomes

Variable	Overall (*n* = 209)
Acute procedural success, *n* (%)	209 (100)
Acute appendage seal, *n* (%)	209 (100)
Total procedure time, min	57.3 ± 17.0
Left atrial dwell time, min	45.1 ± 13.6
Fluoroscopy time (LAAC segment), min	3.4 ± 0.8
Contrast volume, mL	20 ± 5
Same-day discharge, *n* (%)	209 (100)
Single LAAC device used, *n* (%)	196 (94.0)
PFA system and mapping platform, *n* (%)	
Farapulse (Boston Scientific) + Opal HDx	94 (45)
PulseSelect (Medtronic) + EnSite X	46 (22)
Sphere-9/Affera (Medtronic) + Prism	69 (33)
LAAC device type: WATCHMAN FLX Pro, *n* (%)	209 (100)
LAAC device size distribution, *n* (%)	
20 mm	17 (8)
24 mm	46 (22)
27 mm	79 (38)
31 mm	31 (15)
35 mm	25 (12)
40 mm	10 (5)
Additional ablation beyond PVI (PWI, mitral isthmus, CTI, or SVC), *n* (%)	96 (46)

Values are presented as mean ± SD or *n* (%). CT, computed tomography; CTI, cavotricuspid isthmus; ICE, intracardiac echocardiography; LAAC, left atrial appendage closure; PVI, pulmonary vein isolation; PWI, posterior wall isolation; SVC, superior vena cava; TEE, transoesophageal echocardiography. Pre-procedural CT imaging with AI-based device modelling was performed in 144 patients (69%); the remaining patients underwent intraprocedural sizing using TEE due to contrast avoidance in the setting of chronic kidney disease.

### Periprocedural and early safety outcomes

There were no cases of pericardial effusion or tamponade, phrenic nerve injury, AKI, or clinically apparent oesophageal injury. No strokes or transient ischaemic attacks occurred during the index hospitalization or within 30 days post-procedure. Minor access-site bleeding occurred in two patients, and there were no major vascular complications requiring intervention. Additional safety outcomes are summarized in *Table 3*.

**Table 3:** Safety outcomes (in-hospital and within 30 days)

Outcome	Overall (*n* = 209)
Pericardial effusion/tamponade	0 (0)
Stroke	0 (0)
Transient ischaemic attack	0 (0)
Death	0 (0)
Device embolization	0 (0)
Phrenic nerve injury	0 (0)
Oesophageal injury	0 (0)
Acute kidney injury	0 (0)
Major bleeding	0 (0)
Major vascular complication requiring intervention	0 (0)
Minor access-site bleeding/haematoma (conservative management)	2 (1.0)
30 day readmission	0 (0)

Values are presented as *n* (%). Major bleeding was defined as bleeding associated with haemodynamic compromise, transfusion, procedural intervention, a decrease in haemoglobin ≥2 g/dL, bleeding at a critical site, or prolonged hospitalization.

### Follow-up imaging outcomes

Follow-up imaging with CT or TEE was performed at a mean of approximately 112 days following the index procedure, with CT used in 80% of patients for peri-device leak assessment. No patients demonstrated peri-device leak >2 mm. Small peri-device leak (<3 mm) was observed in 4.7% of patients, and there were no moderate or large leaks. Out of the 209 patients, 207 (99%) were transitioned to single antiplatelet therapy; 89% received aspirin and 11% received clopidogrel. Device-related thrombus (DRT) was identified in two patients (1.0%) at scheduled follow-up imaging on Days 84 and 89 post-procedure, respectively, without associated clinical thromboembolic events. In both cases, full-dose OAC was continued, and repeat CT performed approximately 6 weeks later confirmed DRT resolution, after which patients were transitioned to low-dose OAC. Imaging outcomes are summarized in *Table 4*.

**Table 4:** Follow-up imaging and rhythm outcomes

Outcome	Overall (*n* = 209)
Imaging follow-up performed, *n* (%)	209 (100)
Follow-up modality, *n* (%)	
CT	167 (80)
TEE	42 (20)
Time to follow-up imaging, days	111.6 ± 16.5
Peri-device leak, *n* (%)	
None/ ≤ 2 mm	199 (95.3)
Small leak (<3 mm)	10 (4.7)
Moderate/large leak (≥3 mm)	0 (0)
DRT, *n* (%)	2 (1.0)
Rhythm follow-up performed, *n* (%)	209 (100)
Monitoring modality, *n* (%)	
ILR	150 (72)
Intermittent monitoring	59 (28)
Early arrhythmia recurrence after 60-day blanking, *n* (%)	2 (1.0)
Repeat ablation/cardioversion/antiarrhythmic drug escalation, *n* (%)	2 (1.0)

Values are presented as mean ± SD or *n* (%). Arrhythmia recurrence was defined as any documented atrial fibrillation, atrial flutter, or atrial tachycardia episode lasting ≥30 s occurring after a 60 day blanking period. CT, computed tomography; DRT, device-related thrombus; ILR, implantable loop recorder; TEE, transoesophageal echocardiography.

### Arrhythmia-related outcomes

Clinical follow-up and rhythm monitoring were performed according to standard practice. Mean follow-up duration was 111.6 ± 16.5 days. After a 60-day blanking period, arrhythmia recurrence occurred in two patients (1.0%), both in the persistent AF subgroup. Monitoring modalities included implantable loop recorder–based surveillance in 72% of patients, with 28% undergoing intermittent monitoring (e.g. electrocardiogram or ambulatory monitoring). No conclusions regarding durable rhythm control can be drawn from the present follow-up duration.

## Discussion

In this prospective single-centre cohort of 209 consecutive patients undergoing concomitant PFA and LAAC, we demonstrate that a standardized integrated workflow is feasible, highly efficient, and associated with excellent acute procedural success and favourable early safety and device performance. Acute procedural success was achieved in 100% of cases, total procedure time was 57.3 ± 17.0 min with minimal fluoroscopy limited to the LAAC portion (3.4 ± 0.8 min), and same-day discharge was achieved in all patients. Early safety outcomes were notable for the absence of pericardial effusion/tamponade, phrenic nerve injury, AKI, clinically apparent oesophageal injury, or stroke/transient ischaemic attack. Follow-up imaging at approximately 112 days demonstrated no peri-device leaks greater than 2 mm, a low rate of small peri-device leak (4.7%), and rare DRT (two patients) without associated thromboembolic events. Rhythm observations were also favourable at early follow-up, with arrhythmia recurrence observed in only two patients, both in the persistent AF subgroup.

### Integrated rhythm control and stroke prevention in AF: rationale for concomitant therapy

Patients referred for AF ablation frequently share clinical characteristics that confer elevated stroke risk and bleeding vulnerability, creating a practical overlap between those eligible for rhythm control interventions and those considered for LAAC. Contemporary AF management frameworks emphasize integrated, patient-centred care, including rhythm control strategies when clinically appropriate alongside individualized stroke prevention approaches.^1^ In parallel, LAAC has emerged as an evidence-based alternative to long-term OAC for stroke prevention in selected patients with non-valvular AF, supported by randomized trial data compared with warfarin and direct OACs.^3,4^ In this context, performing ablation and LAAC concomitantly has intuitive advantages, including avoidance of staged transseptal procedures, reduced cumulative anaesthesia exposure, shortened total hospitalization time, and potentially improved patient experience. However, adoption of combined strategies has been limited historically by concerns regarding incremental periprocedural risk, the possibility of device seating/seal challenges in the setting of acute post-ablation atrial oedema, and uncertainty regarding downstream device-related outcomes.

### Procedural efficiency and the role of PFA

A key finding in this study is that concomitant therapy can be delivered with procedural times comparable to those of standalone ablation, suggesting that integration does not necessarily translate into excessive complexity when performed within a standardized workflow. In addition to a single transseptal strategy supported by ICE and EAM, pre-procedural CT planning with AI-based assessment likely contributed to procedural streamlining and consistency of LAAC delivery. Although this study was not designed to independently assess the procedural impact of AI-based planning, prior studies have demonstrated that FEops HEARTguide planning is associated with reduced procedure time, increased rates of single-device implantation, and lower fluoroscopy exposure during LAAC procedures. (De Backer) Accordingly, the incorporation of AI-based planning in this workflow reflects established institutional practice supported by existing evidence. This is particularly relevant as PFA expands globally. PFA has been shown to be noninferior to thermal ablation for paroxysmal AF and has demonstrated favourable safety outcomes in pivotal studies, supporting its suitability as a platform for integrated procedural care models.^6,7,9,10^ The present data suggest that leveraging PFA within a combined procedure may allow efficient delivery of both rhythm control and stroke prevention in a single setting while maintaining a favourable safety profile. Given the short follow-up duration, non-uniform rhythm monitoring, and application of a 60 day blanking period, rhythm outcomes in this study should be considered exploratory and should not be compared with long-term ablation efficacy trials.

### Device performance and imaging outcomes following concomitant PFA + LAAC

Device-related endpoints are a central concern in concomitant ablation and LAAC, particularly given the theoretical risk that acute oedema or altered appendage geometry could influence compression, seal, or DRT. In this cohort, acute appendage seal was achieved in all patients, and follow-up imaging at approximately 112 days demonstrated no peri-device leaks greater than 2 mm and a low rate of small peri-device leak (4.7%). Notably, CT imaging was used for follow-up assessment in 80% of patients, supporting rigorous characterization of peri-device leak in this cohort. Although two cases of DRT were observed, neither was associated with thromboembolic events, and both were managed with extension of anticoagulation therapy. Both DRT cases resolved on follow-up imaging with continuation of anticoagulation and subsequent transition to low-dose OAC. The observed 1.0% DRT rate is numerically consistent with early post-implantation DRT rates reported in contemporary WATCHMAN FLX studies, although direct comparison is limited by the short follow-up duration of the present analysis.^11–13^ In addition, large registry data suggest that residual peri-device leak carries prognostic relevance, supporting rigorous imaging assessment and standardized follow-up strategies.^14^ Extended lesion sets (PW, mitral isthmus, CTI, and/or SVC) may increase procedural duration and acute atrial oedema and could theoretically influence LAA geometry, device sizing, or early seal assessment. Accordingly, the early device outcomes observed in this cohort should be interpreted in the context of heterogeneous ablation extent. Future analyses stratified by PVI-only vs. extended ablation will be important to determine whether ablation extent modifies procedural efficiency or LAAC-related imaging outcomes.

### Comparison with prior data and contribution of the present study

Available data on concomitant PFA and LAAC remain limited. A recent single-centre experience reported feasibility and favourable early outcomes with concomitant PFA and LAAC, demonstrating high acute procedural success and low rates of periprocedural complications.^5^ Our study extends these observations substantially by evaluating a standardized integrated workflow in a much larger consecutive cohort (*n* = 209), demonstrating marked procedural efficiency with minimal fluoroscopy exposure and universal same-day discharge, alongside excellent early safety and device performance. Importantly, concomitant ablation and LAAC has also been reported in prior studies performed with thermal ablation modalities, demonstrating feasibility and acceptable outcomes and providing a foundation for contemporary integrated strategies.^15–17^

### Implications

These results have several implications for clinical practice and programme design. First, they support a ‘one-stop’ care model in which rhythm control and stroke prevention can be offered during the same procedural encounter for appropriately selected high-risk patients. Second, they demonstrate that when performed within a standardized approach, the combined workflow need not increase procedural complexity to a prohibitive degree; indeed, procedural duration and fluoroscopy exposure remained low. Finally, the favourable safety profile observed in this large consecutive series supports continued exploration of integrated EP-structural strategies, particularly as PFA adoption expands and procedural efficiency becomes increasingly important in high-volume AF programmes.

### Limitations

This study should be interpreted considering several limitations. It represents the experience of a single centre and reflects procedural expertise and workflow optimization that may not be generalizable to all settings. Follow-up duration is relatively short (∼112 days), limiting assessment of long-term rhythm outcomes, device durability, and late complications, including late DRT or delayed peri-device leak changes. Rhythm monitoring strategies were not uniform, although most patients underwent implantable loop recorder surveillance, which may influence recurrence ascertainment compared with intermittent monitoring approaches. The absence of a staged-procedure or standalone ablation/LAAC comparator group limits direct comparative assessment, and this study was not designed to evaluate the relative efficacy or safety of concomitant vs. staged strategies. All cases occurred in a highly experienced and large volume centre with standardized CT-based AI planning, universal EAM use, and universal same-day discharge. This limits reproducibility of these results in lower-volume centres or with less experienced operators.

## Conclusions

In a large consecutive cohort, a standardized concomitant workflow for PFA and LAAC was feasible and highly efficient, with 100% acute procedural success, minimal fluoroscopy exposure, and universal same-day discharge. Early safety outcomes were favourable, and follow-up imaging demonstrated excellent device performance with no peri-device leaks greater than 2 mm and rare DRT. Early favourable rhythm observations during limited follow-up should be interpreted cautiously and require longer-term assessment. Longer-term follow-up would also be required to confirm durability and late device-related outcomes. These findings support concomitant PFA and LAAC as a practical integrated strategy for delivering rhythm control and stroke prevention in appropriately selected patients with AF.

### Clinical implications

- Concomitant PFA and LAAC can be performed safely and efficiently using a standardized workflow, enabling integrated rhythm control and stroke prevention in a single procedural encounter.
- A minimal fluoroscopy strategy confined to the LAAC segment, combined with ICE/EAM guidance and CT-based planning, may facilitate procedural efficiency, and support same-day discharge pathways.
- Excellent early imaging outcomes and low DRT rates suggest that concomitant therapy does not compromise early LAAC performance when performed within a consistent sizing and imaging protocol.

### Future directions

- Larger multi-centre studies with longer follow-up are needed to confirm long-term arrhythmia outcomes, late DRT, and durability of peri-device seal following concomitant PFA and LAAC.
- Comparative studies evaluating concomitant vs. staged strategies may clarify optimal patient selection, anticoagulation strategies, and resource utilization within integrated AF care models.

### Key points

- In 209 consecutive patients, a standardized concomitant PFA + LAAC workflow achieved 100% acute procedural success with 57.3 ± 17.0 min total procedure time and 3.4 ± 0.8 min fluoroscopy time.
- Same-day discharge was achieved in 100% of patients, supporting an efficient integrated care pathway.
- Early safety outcomes were favourable, with no pericardial effusion/tamponade, phrenic nerve injury, AKI, oesophageal injury, or stroke/TIA (in-hospital + 30 days).
- Follow-up imaging at ∼112 days demonstrated no peri-device leaks >2 mm, with 4.7% small leaks and rare DRT (two patients).
- Early arrhythmia recurrence after a 60 day blanking period was low (two patients, both persistent AF), though longer follow-up is required.
